# Effects of Energy and Protein Levels on Laying Performance, Egg Quality, Blood Parameters, Blood Biochemistry, and Apparent Total Tract Digestibility on Laying Hens in an Aviary System

**DOI:** 10.3390/ani12243513

**Published:** 2022-12-12

**Authors:** Chan-Ho Kim, Hwan-Ku Kang

**Affiliations:** 1 Animal Welfare Research Team, National Institute of Animal Science, Rural Development Administration, Wanju 55365, Republic of Korea; 2 Poultry Research Institute, Rural Development Administration National Institute of Animal Science, Pyeongchang 25342, Republic of Korea

**Keywords:** aviary system, laying hens, apparent metabolizable energy, protein, nutrient

## Abstract

**Simple Summary:**

The diet’s energy and protein content must be adjusted to ensure that hens consume enough nutrients needed to cope with growth and onset of egg production. However, the main difference in nutrition between conventional cage and alternative housing is the energy requirements. Laying hens in alternative production systems are more active. Energy requirements for activity depends on the type of system and is therefore higher in alternative and free range systems.

**Abstract:**

This study was performed to investigate the effects of apparent metabolizable energy (AME_n_) and protein levels on laying performance, egg quality, blood parameters, blood biochemistry, and apparent total tract digestibility of energy and nutrients in diets fed to laying hens in an aviary system. A total of 560 Hy-Line Brown laying hens (age = 30 week) were distributed in a completely randomized experimental design in 2 × 2 factorial arrangements with 2 metabolizable energy levels (2700 and 2800 kcal AME_n_/kg) and 2 protein levels (16.5 and 14.5% CP). Four treatments and four replicates of 40 birds each (stocking density = 15 birds/m^2^) were prepared. Results revealed no significant interaction between AME_n_ and CP in the diet in terms of egg production, floor eggs, broken and dirty egg production, egg mass, feed intake, and feed conversion ratio of laying hens. However, egg weight was affected. As dietary energy and CP levels (2800 kcal of AME_n_/kg and 16.5% CP) increased, egg weight increased (*p* < 0.05). Egg weight, feed intake, and feed conversion ratio significantly differed (*p* < 0.05) as the energy content in the feed increased. Ether extract significantly varied (*p* < 0.05) as the energy content in the feed increased. In conclusion, laying performance and egg quality of Hy-Line Brown laying hens in the middle stage of egg production (30 to 50 weeks) were not affected by different dietary energy and protein levels, but feed intake decreased with an increasing level of AME_n_ in diets. Ether extract significantly varied as the energy content in the feed increased.

## 1. Introduction

Aviary systems have been identified as possible alternatives to cages, providing the hens with a larger total available area and access to nests, litter and perches [1,2]. Traditionally, conventional laying houses are made for domestic chickens in Korea; therefore, nutrition, management, egg quality, and safety of conventional breeding systems have been comprehensively explored. In Korea, public research on production, egg quality, and nutrition has been limited to layer farms, an alternative breeding system, and the combined effects of protein levels and energy levels on laying hens in aviary system performance have been rarely investigated. The 32-to-45-week laying period energy concentration and protein levels have been estimated to be 2700 kcal of apparent metabolizable energy (AME_n_)/kg and 16.5%, respectively [3]. However, non-cage breeding is expected to increase energy demand because of increased activity. Various dietary energy levels (2684–2992 kcal of metabolizable energy) are also used in the egg industry. However, information concerning the ideal dietary energy level required for optimal laying performance is limited [4]. Hens generally adjust their feed intake according to their energy requirements. Harms et al. [5] and Leeson et al. [6] showed that the hen-day egg production is not influenced by dietary energy levels. However, when energy intake is deficient, egg production declines. Harms et al. [5] also observed that hens fed with increased energy diets produce heavier eggs. According to NRC [7], diets for laying hens may be formulated with minimal protein levels to meet the nitrogen requirements for the synthesis of non-essential and essential amino acids. Leeson et al. [6] also indicated that laying hen diets are generally formulated with excess protein which increases nitrogen loss due to excretion. Bhatti and Sharma [8] observed that egg weight and hen-day egg production decrease, and mortality increases when dietary protein is decreased from 17% to 13%. Medonca and Lima [9] showed that a decrease in protein levels from 16.5% to 14.5% causes a significant decline in eggshell quality at the beginning of the second production cycle. In this study, the following hypothesis was tested: an interaction between AME_n_ and CP concentrations in the diet could improve the laying production performance of laying hens in an aviary system. To the best of our knowledge, limited research has been published on the interaction of AME_n_ and CP concentrations in the diet on laying performance, egg quality, blood parameters, blood biochemistry values, and apparent total tract digestibility (ATTD) of energy and nutrients in diets fed to laying hens in an aviary system.

## 2. Materials and Methods

The protocol for this experiment was reviewed and approved by the Institutional Animal Care and Welfare Committee of the National Institute of Animal Science, Rural Development Administration, Republic of Korea (NIAS 2019-364).

### 2.1. The Aviary System

The aviary, a modified Netherlands system (Comfort 2 Aviary System, Jansen, NLD), was divided into 12 pens by using wire netting walls. The hens were housed in 16 identical pens of an aviary system composed of litter (rice hulls), single nests, and three welded wire tiers with nipple drinkers. Nests in multi-tiers with automatic egg collection belts were attached to the walls of the room opposite the aviary tiers.

### 2.2. Bird and Experimental Design

A total of 560 Hy-Line Brown laying hens (*Gallus gallus domesticus*) were used. They were reared from 1 d of age under the same conditions with feed and water supplied on raised slatted floors with high perches. From week five, they were given access to litter between the slatted areas. At 14 weeks, the laying hens were transferred to a laying house. 30-week-old Hy-Line Brown laying hens were distributed in a completely randomized experimental design in a 2 × 2 factorial arrangement with two apparent metabolizable energy levels (2700 and 2800 kcal of AME_n_/kg) and two protein levels (16.5 and 14.5% CP) (Table 1). Four treatments and four replicates of 40 birds each (stocking density of 15 birds/m^2^; pen size = 1.7 m × 1.57 m × 2.7 m; water nipple = 7; n = 40) were prepared. Diets based on corn and soybean meal were made of equal levels of calcium (3.90%) and phosphorus (0.31% available P) and balanced in accordance with KFSP [3] recommendations. During the 20-week experimental period, the hens were provided with feed and water *ad libitum* and exposed to a 16 h:8 h (light: dark) schedule. The temperature and humidity of the laying house were maintained at 20 ± 3 °C and 65–70%, respectively. The diets were analyzed in triplicate to determine the crude protein (CP), crude ash, ether extract, and gross energy (GE) contents. Nitrogen for CP analysis was measured using a nitrogen analyzer (NS-2000, Leco Corp., St. Joseph, MI, USA). Dry matter was determined in accordance with the AOAC [10] method, and GE was determined using a Parr adiabatic oxygen bomb calorimeter (Parr Instrument CO., Moline, IL, USA). Ether extracts of the diet samples were analyzed [10].

### 2.3. Laying Perfomance Parameters

To access egg productivity, egg production (hen-day and hen house), mean egg weight, floor egg rate, broken and shell-less egg productions were recorded daily and used to calculate a weekly average. The daily feed intake was measured weekly and the feed conversion ratio (the ratio of feed intake to egg mass, g/g) was calculated.

### 2.4. Determination of Egg Quality Parameters

At the end of the experiments, 50 eggs per treatment were randomly collected. Measurement of egg weight, albumen height, Haugh unit (HU), and yolk color were conducted using an egg multi-tester (EA-01 type, ORKA Food Technology Ltd., Ramat Hasharon, Israel). The strength of the eggshell was measured at the precise moment the shell broke using an eggshell force gauge (Eggshell Force Gauge Model II, Robotmation Co., Ltd., Tokyo, Japan) by gradually increasing the pressure strength of the horizontally fixed egg to its breaking point. The eggshell thickness is defined as the mean value of measurements at three different locations on the egg (air cell, equator, and sharp end) and was measured using a caliper (Digimatic Micrometer, Series 547-360, Mututoyo, Japan) after the eggshell membrane was removed.

### 2.5. Hematological Analysis

At the end of the experiment period, the blood samples were collected, using EDTA-treated BD Vacutainer^®^ tubes (Becton Dickinson, Franklin Lakes, NJ, USA), from the wing vein of 10 birds randomly selected from each treatment. The tubes were placed on ice, and the whole-blood samples were immediately evaluated. Leukocyte (white blood cells, heterophils, lymphocytes, monocytes, eosinophils, and basophils) from the blood samples were analyzed using a Hemavet Multispecies Hematology System (Drew Scientific Inc., Oxford, CT, USA). The H/L ratio was calculated as lymphocytes divided by heterophils. Then, the samples were centrifuged at 25,000× *g* and 4 °C for 20 min to obtain the sera that were then stored at −15 °C. Total cholesterol, triglyceride, aspartate aminotransferase (AST), alanine aminotransferase (ALT), and calcium in the sera were quantified using ADVIA 1650 Chemistry System (Bayer Diagnostic, Puteaux, France).

### 2.6. Nutrient Digestibility

The hens were fed with the experimental diets for 8 d and subjected to 5 d of adaptation. Afterward, their excreta were collected for 3 d. The marker versus the marker method was used to securely collect waste excreted from the hens [11]. Chromium oxide (0.3%) and ferric oxide (0.3%) were added to the feed at the beginning and end of the collection period. Excretion collection was initiated when chromium oxide was detected in the excreta and then terminated when iron oxide was found in the excreta. The procedure was carefully performed to prevent the hens from interfering with fecal monitoring. Food intake was recorded, and excreta were collected daily and stored at −4 °C for analysis. The excretory samples were dried in a forced-air oven at 60 °C for 72 h and finely ground for subsequent analysis. The diet and excretory samples were analyzed for GE using a bomb calorimeter (Parr Instrument). The feed and excretory samples were analyzed for dry matter (method 934.01), CP (method 990.93), ether extract (method 920.39), and crude ash (method 942.05) in accordance with the standard procedure of the AOAC [10]. The dietary GE and nutrient ATTD were calculated as 100 − (total GE or nutrient excretion/total GE or nutrient intake) × 100 [12].

### 2.7. Statistical Analysis

Data were analyzed using a completely randomized design via two-way ANOVA in accordance with the MIXED procedure of SAS [13]; in this design, the replicate was set as the experimental unit. Outlier data were checked via the UNIVARIATE procedure of SAS [14], but no outliers were identified. The following statistical model was used: *Y_ijk_* = *μ* + *E_i_* + *P_j_* + *EP_ij_* + *e_ijk_*, where *Y_ijk_* is the individual observation, *μ* is the overall mean, *E_i_* is the effect of dietary energy, *P_j_* is the effect of the dietary protein level, *EP_ij_* is the effect of interaction, and *e*_ijk_ is the random error. An alpha level of 0.05 was used to determine statistical significance. When the model was significant, Tukey’s test was performed to make pairwise comparisons between the mean values. Data were presented as least-squares means and SEM.

## 3. Results

### 3.1. Laying Performance and Eggshell Quality

AME_n_ and CP in the diet had no significant interaction in terms of hen-day production, hen house production, mortality, floor eggs, broken and dirty egg production, feed intake, and feed conversion ratio of laying hens (Table 2), but egg weight was affected. Specifically, as dietary energy and CP levels (2800 kcal of AME_n_/kg and 16.5% CP) increased, egg weight increased (*p* < 0.05) from 62.4 to 64.5g. However, egg weight, feed intake, and feed conversion ratio significantly differed (*p* < 0.05) as the energy content in the feed increased. In addition, the increase in CP levels in the diets did not influence hen-day egg production egg weight, floor eggs, broken and dirty eggs, egg mass, feed intake, and feed conversion ratio. There were no significant interactions between AME_n_ and CP in the diet affecting eggshell strength, eggshell thickness, egg yolk color, or Haugh unit of the laying hens (Table 3) which were also not influenced by the increase in AME_n_ and CP levels in the diets.

### 3.2. Hematological Analysis

For leukocyte (WBC, HE, LY, MO, EO, and BA) and erythrocyte (RBC, Hb, HCT, MCV, MCH, MCHC) concentration did not affect significant interactions between AME_n_ and CP in the diet (Table 4 and Table 5). The interaction between AME_n_ and CP in the diet did not affect blood biochemistry values (total cholesterol, triglyceride, total protein, AST, ALT, and calcium; Table 6).

### 3.3. Nutrient Digestibility

The nutrient digestibility results are summarized in Table 7. Gross energy, dry matter, crude protein, ether extract, and crude ash did not reflect significant interactions between AME_n_ and CP in the diet. However, ether extract significantly varied (*p* < 0.05) as the energy content in the feed increased.

## 4. Discussion

Nutritional concentration of feed is very important in the poultry industry as it affects both laying performance and profitability. Yu et al. [15] reported that egg production, egg weight, and egg mass are higher in hens fed with diets containing 2800 kcal/kg AME_n_/kg than in hens fed with diets comprising 3080 and 3360 kcal AME/kg. In the present experiment, the dietary interaction between AME_n_ and CP significantly affected egg weight. Specifically, egg weight increased as dietary energy and protein increased. Yu et al. [15] and Gunawardana et al. [16] did not observe any effect of energy and protein interaction on egg weight, but they found that egg weight increases with CP in a concentration-dependent manner. Consistent with [4,17,18], the present experiment demonstrated that the feed intake decreased by 3.45% when AME_n_ levels in the diet increased from 2650 kcal/kg to 2750 kcal/kg. Therefore, supplemental fat often increases egg weight [19,20,21]. Hens eat to satisfy their energy requirements; therefore, an increase in dietary energy content should decrease their feed intake [22]. However, an increase in the current energy content of the diet from 2700 kcal of AME_n_/kg to 2800 kcal of AME_n_/kg (a 3.57% increase) resulted in a decreased feed intake of 4.83%. Dietary energy content can be increased by increasing the amount of fat; supplemental fat often results in an increased energy intake probably because of less dust formation and improved palatability of the diet [23]. Harms et al. (2000) observed no significant differences in hen-day egg production of Brown hens and Single Comb White Leghorns (SCWL) fed with diets containing varying AME from 2500 kcal/kg to 3100 kcal/kg as the energy content of the diet changes. These data support the hypothesis that excess energy intake caused by changes in dietary composition primarily increases body weight gain rather than egg mass production. Hens fed with a high AME_n_ diet (2800 kcal/kg in the current experiment) tend to overconsume energy that positively affects their feed intake and feed conversion ratio but not egg production or egg mass. Increasing dietary protein intake from 13.8 to 17.1 g/hen per day increased egg mass 5.75 g/hen per day and egg weight by 2.38 g, respectively. The mechanism by which protein improves egg size is well understood [24]. Yolk color has a considerable influence on the marketability of eggs; the color of the yolk depends on the fat-soluble carotenoids and xanthophylls [16,17]. We observed that the yolk color increased with the increase of the dietary energy level. Similar findings were reported by Perez Bonilla et al. [17] and Xin et al. [25], who found that yolk pigmentation increased linearly with an increase in energy concentration of the diet even though all diets had similar levels of pigmenting additives and corn. An earlier report that increasing the energy level of the diet from 2850 kcal/kg to 3050 kcal/kg did not influence the eggshell quality and Haugh unit [26]. Similarly, Kang et al. [22] reported that eggshell thickness, eggshell strength, and Haugh unit were not influenced by inclusion of energy and nutrient density diets (2700 or 2800 kcal/kg AME_n_). It has been shown that blood total cholesterol moves in the opposite direction with the level of albumen and triglyceride content. Possible reasons for this might be due to the ingredients (soybean meal and corn) used in the diets. This result is consistent with previous studies which reported that different energy and protein levels did not affect blood components [27]. Blood parameters are good indicators of general health status, reflecting any physiological, nutritional, and pathological changes occurring in organisms. Leukocyte count has also been used as a measure of immune function in birds [28]. However, the role of the interaction between AME_n_ and CP in altering blood parameters in poultry should be further investigated. Previous studies reported that the inclusion of dietary fat increases the retention time of the digesta in chickens [29]. The observation for increased transit time of birds by feeding them a diet containing tallow in this experiment agrees with previous reports that inclusion of dietary fat increases retention time of digesta in chickens [29,30]. Accordingly, increased digesta transit time may be the reason for increased utilization of DM, GE, CP, and crude fat in diets with increased AMEn as the greater digesta retention time subsequently increases nutrient availability and absorption [31]. Increased ATTD of crude fat in diets containing tallow is also likely caused by the fact that supplemented tallow is more digestible than indigenous forms of fat that are naturally present in feed ingredients (e.g., corn, and soybean meal) used for the basal diet [32]. Fat digestibility results obtained in this experiment for the middle stage of egg production are in agreement with those of Tokach et al. [33]. Lawrence et al. [34] and Smith et al. [35] reported that dietary energy level affected various nutritional responses. Energy intake is known to influence protein deposition [36]. Earlier reports indicate that the amount of energy in feed could change N-retention to increase protein digestibility [34,35]. The results in this study are similar to those of Van Lunen and Cole [36] that decreasing energy density could reduce protein and lipid gain.

## 5. Conclusions

Laying performance and egg quality of Hy-Line Brown laying hens in the middle stage of egg production (30 to 50 weeks) were not affected by different dietary energy and protein levels, but feed intake decreased with an increasing level of AME_n_ in their diets. Ether extract significantly varied as the energy content in the feed increased. A diet with 2800 kcal of AMEn/kg for laying hens is adequate for satisfactory ether extract digestibility.

## Figures and Tables

**Table 1:** Composition and nutrient content of experimental diets.

Ingredients, %	2800 kcal of AME/kg Protein Levels (%)		2700 kcal of AME/kg Protein Levels (%)	
	16.5	14.5	16.5	14.5
Corn	60.90	67.09	58.73	64.42
Soybean meal	24.55	19.25	21.32	16.10
DDGS	2.17	2.17	8.17	8.17
Soybean oil	1.30	0.37	0.53	-
Limestone	9.63	9.67	9.89	9.92
MDCP	0.81	0.81	0.76	0.79
99%_D,L_-methionine	0.19	0.19	0.15	0.15
Sodium chloride	0.25	0.25	0.25	0.25
Sodium bicarbonate	0.20	0.20	0.20	0.20
Vitamin premix ^1^	0.10	0.10	0.10	0.10
Mineral premix ^2^	0.10	0.10	0.10	0.10
Total	100.00	100.00	100.00	100.00
Calculated composition ^3^				
ME, kcal/kg	2800	2800	2700	2700
Crude protein, %	16.5	14.5	16.5	14.5
Calcium, %	3.90	3.90	3.90	3.90
Available phosphate, %	0.31	0.31	0.31	0.31
Lysine, %	0.85	0.85	0.85	0.85
Methionine + Cystein, %	0.71	0.71	0.71	0.71
Analyzed composition ^4^				
Gross energy, kcal/kg	3610	3617	3449	3465
Dry matter, %	89.63	88.85	90.23	89.54
Crude protein, %	16.22	14.61	16.38	14.32
Crude fat, %	3.65	3.20	3.48	3.08

^1^ Provided per kilogram of the complete diet: vitamin A (vitamin A acetate), 12,500 IU; vitamin D_3_, 2500 IU; vitamin E (DL- α-tocopheryl acetate), 20 IU; vitamin K_3_, 2 mg; vitamin B1, 2 mg; vitamin B_1_, 2mg; vitamin B_2_, 5 mg; vitamin B_6_, 3 mg; vitamin B_12_, 18 μg; calcium pantotenate, 8 mg; folic acid, 1 mg; biotin 50 μg; niacin, 24 mg. ^2^ Provided per kilogram of the complete diet: Fe (FeSO_4_·7H_2_O), 40 mg; Cu (CuSO_4_·H2O), 8 mg; Zn (ZnSO_4_·H_2_O), 60 mg; Mn(MnSO_4_·H_2_O), 90 mg; Mg (MgO) as 1500 mg. ^3^ Nutrient contents in all diet were calculated. ^4^ Triplicate Analysis.

**Table 2:** Laying performance characteristics of hens fed with different AME_n_ and CP levels in an aviary system ^1^.

Items		Hen-Day Production, %	Hen House Production, %	Mortality, %	Egg Weight, g	Floor Eggs, %	Broken & Dirty Eggs, %	Feed Intake (g/Bird/Day)	FCR
AME_n_, kcal/kg	CP, %								
2800	16.5	90.09	88.58	1.00	64.49 ^a^	2.99	1.60	130.5	2.29
2800	14.5	88.53	88.20	0.30	63.31 ^b^	3.43	1.66	129.6	2.27
2700	16.5	89.48	88.50	0.65	62.38 ^c^	3.05	1.43	135.9	2.44
2700	14.5	88.30	87.98	0.68	62.37 ^c^	2.05	1.57	137.3	2.49
SEM ^2^		1.163	1.145	0.025	0.189	1.530	0.117	0.727	0.039
AME_n_, kcal/kg									
2800		89.32	88.39	0.65	63.90 ^a^	3.21	1.63	130.0 ^b^	2.28 ^b^
2700		88.89	88.24	0.67	62.38 ^b^	2.55	1.50	136.6 ^a^	2.46 ^a^
SEM ^2^		0.822	0.798	0.018	0.133	1.082	0.082	0.514	0.028
CP, %									
16.5		89.78	88.54	0.83	63.30	3.24	1.61	133.2	2.36
14.5		88.42	88.09	0.49	63.43	2.52	1.52	133.5	2.38
SEM ^2^		0.822	0.798	0.018	0.133	1.082	0.082	0.514	0.028
Effects (*p*-value)									
AME_n_		0.721	0.719	0.258	0.002	0.683	0.321	0.004	0.005
CP		0.872	0.826	0.697	0.152	0.655	0.422	0.687	0.591
AME_n_ × CP		0.271	0.256	0.325	0.012	0.867	0.744	0.156	0.402

^a–c^ Means in the same row with different superscripts differ significantly (*p* < 0.05). ^1^ Data are least squares means of 5 replicates per treatment. ^2^ Standard error of means.

**Table 3:** Egg quality characteristics of hens fed with different AME_n_ and CP levels in an aviary system ^1^.

Items		Eggshell Strength, kg/cm^2^	Eggshell Thickness, μm	Egg Yolk Color	Haugh Unit
AME_n_, kcal/kg	CP, %				
2800	16.5	3.81	397.3	5.76	86.0
2800	14.5	3.40	392.7	6.27	89.3
2700	16.5	3.90	404.0	6.02	84.2
2700	14.5	4.13	412.3	5.12	83.2
SEM ^2^		0.201	6.000	0.384	0.187
AME_n_, kcal/kg					
2800		3.60	395.0	6.01	87.6
2700		4.01	408.2	5.57	83.7
SEM ^2^		0.142	4.242	0.271	1.285
CP, %					
16.5		3.86	400.7	5.89	85.1
14.5		3.76	402.5	5.70	86.3
SEM ^2^		0.142	4.242	0.271	1.285
Effects (*p*-value)					
AME_n_		0.325	0.304	0.875	0.312
CP		0.654	0.767	0.292	0.523
AME_n_ × CP		0.121	0.283	0.331	0.237

^1^ Data are least squares means of 50 replicates per treatment. ^2^ Standard error of means.

**Table 4:** Leukocyte characteristics of hens fed with different AME_n_ and CP levels in an aviary system ^1^.

Items		Leukocyte ^3^					
		WBC, K/µL	HE, K/µL	LY, K/µL	MO, K/µL	EO, K/µL	BA, K/µL
AME_n_, kcal/kg	CP, %						
2800	16.5	10.04	1.22	7.86	0.88	0.07	0.01
2800	14.5	13.96	2.43	10.03	1.15	0.11	0.02
2700	16.5	11.80	1.63	8.77	0.93	0.06	0.01
2700	14.5	11.32	1.69	8.39	1.09	0.12	0.02
SEM ^2^		1.482	0.430	0.872	0.230	0.047	0.008
AME_n_, kcal/kg							
2800		12.00	1.83	8.94	1.02	0.09	0.01
2700		11.56	1.66	8.58	1.01	0.09	0.02
SEM ^2^		1.048	0.304	0.622	0.162	0.033	0.059
CP, %							
16.5		10.92	2.06	8.32	0.91	0.06	0.01
14.5		12.64	1.43	9.21	1.12	0.12	0.02
SEM ^2^		1.048	0.304	0.622	0.162	0.033	0.059
Effects (*p*-value)							
AME_n_		0.131	0.771	0.717	0.981	0.944	0.681
CP		0.712	0.262	0.162	0.363	0.283	0.222
AME_n_ × CP		0.644	0.163	0.204	0.849	0.842	0.864

^1^ Data are least squares means of 10 replicates per treatment. ^2^ Standard error of means. ^3^ Leukocytes: WBC; white blood cell, HE; heterophil, LY; lymphocytes, MO; monocyte, EO; eosinophil, BA; basophil.

**Table 5:** Leukocyte characteristics of hens fed with different AME_n_ and CP in an aviary system ^1^.

Items		Erythrocyte ^3^					
		RBC, M/µL	Hb, g/dL	HCT, %	MCV, fL	MCH, pg	MCHC, g/dL
AME_n_, kcal/kg							
2800	2.38	8.28	23.28	97.78	38.18	39.12	0.01
2800	2.46	8.48	22.56	100.06	38.58	38.68	0.02
2700	2.36	8.24	23.60	100.38	38.06	37.92	0.01
2700	2.31	8.66	22.60	97.86	37.52	38.32	0.02
SEM ^2^		0.123	0.270	1.096	1.925	0.765	0.780
AME_n_, kcal/kg							
2800	2.42	8.45	22.92	98.92	38.38	38.90	0.01
2700	2.34	8.38	23.10	99.12	37.79	38.12	0.02
SEM ^2^	0.087	0.191	0.775	1.361	0.541	0.552	0.059
CP, %							
16.5	2.37	8.26	23.44	99.08	38.12	35.52	0.01
14.5	2.39	8.57	22.58	98.96	38.05	38.50	0.02
SEM ^2^	0.087	0.191	0.775	1.361	0.541	0.552	0.059
Effects (*p*-value)							
AME_n_		0.521	0.801	0.878	0.921	0.451	0.332
CP		0.872	0.274	0.441	0.952	0.932	0.987
AME_n_ × CP		0.619	0.687	0.903	0.233	0.554	0.601

^1^ Data are least squares means of 10 replicates per treatment. ^2^ Standard error of means. ^3^ Erythrocytes: RBC; red blood cell, Hb; hemoglobin, HCT; hematocrit, MCV; mean corpuscular volume, MCH; mean corpuscular hemoglobin, MCHC; mean corpuscular hemoglobin concentration.

**Table 6:** Blood biochemistry characteristics of hens fed with different AME_n_ and CP levels during the 30 to 50 weeks in an aviary system ^1^.

Items		Total Cholesterol, mg/dL	Triglyceride, mg/dL	Total Protein, mg/dL	AST, U/L	ALT, U/L	Calcium, mg/dL
AME_n_, kcal/kg	CP, %						
2800	16.5	109.0	1,186.0	5.45	29.70	32.22	32.65
2800	14.5	121.3	1,273.3	6.51	27.90	28.99	32.11
2700	16.5	129.2	1,331.8	4.41	31.73	39.12	27.85
2700	14.5	128.7	1,446.1	5.41	33.17	38.66	33.50
SEM ^2^		10.565	80.873	0.800	2.445	3.183	1.374
AME_n_, kcal/kg							
2800		115.1	1,229.7	5.98	28.80	30.60	32.38
2700		129.0	1389.0	4.91	32.45	30.89	30.68
SEM ^2^		7.470	57.186	0.565	1.729	2.501	0.972
CP, %							
16.5		119.1	1,258.9	4.93	30.72	35.67	30.25
14.5		125.0	1,359.7	5.96	30.54	33.82	32.81
SEM ^2^		7.470	57.186	0.565	1.729	2.501	0.972
Effects (*p*-value)							
AME_n_		0.211	0.641	0.201	0.154	0.131	0.241
CP		0.588	0.232	0.222	0.943	0.572	0.133
AME_n_ × CP		0.551	0.874	0.964	0.522	0.677	0.310

^1^ Data are least squares means of 10 replicates per treatment. ^2^ Standard error of means.

**Table 7:** Nutrient digestibility of hens fed with different AME_n_ and CP levels during the 30 to 50 weeks in an aviary system ^1^.

Items		Gross Energy	Dry Matter	Crude Protein	Ether Extract	Crude Ash
		%				
AME_n_, kcal/kg	CP, %					
2800	16.5	82.99	77.78	58.15	95.64	56.68
2800	14.5	83.21	77.57	57.49	96.05	53.22
2700	16.5	82.95	77.28	58.76	94.00	55.18
2700	14.5	82.01	77.62	57.01	93.20	54.78
SEM ^2^		1.685	1.556	3.267	0.816	4.243
AME_n_, kcal/kg						
2800		83.10	77.67	57.89	95.84 ^a^	54.83
2700		82.48	77.45	57.82	93.60 ^b^	54.94
SEM ^2^		0.859	1.101	2.191	0.577	3.098
CP, %						
16.5		82.97	77.53	58.45	94.82	55.93
14.5		82.61	77.59	57.25	94.62	53.85
SEM ^2^		0.859	1.101	2.191	0.577	3.098
Effects (*p*-value)						
AME_n_		0.365	0.887	0.982	0.001	0.978
CP		0.582	0.968	0.704	0.812	0.638
AME_n_ × CP		0.658	0.863	0.864	0.470	0.755

^1^ Data are least squares means of 5 replicates per treatment. ^2^ Standard error of means.

## Data Availability

Not applicable.
